# *Neisseria meningitidis* accumulate in large organs during meningococcal sepsis

**DOI:** 10.3389/fcimb.2023.1298360

**Published:** 2023-11-28

**Authors:** Berit Sletbakk Brusletto, Bernt Christian Hellerud, Reidun Øvstebø, Petter Brandtzaeg

**Affiliations:** ^1^ Department of Medical Biochemistry, Oslo University Hospital, Oslo, Norway; ^2^ Institute of Immunology, Oslo University Hospital, Oslo, Norway; ^3^ Department of Pediatrics, Oslo University Hospital, Nydalen, Norway; ^4^ Institute of Clinical Medicine, Faculty of Medicine, University of Oslo, Oslo, Norway

**Keywords:** *Neisseria meningitidis*, meningococcal septic shock, LPS (lipopolysaccharide), meningococcal DNA, LAL-assay, multiple organ failure - MOF, porcine shock model, LPS in tissue samples

## Abstract

**Background:**

*Neisseria meningitidis* (Nm) is the cause of epidemic meningitis and fulminant meningococcal septicemia. The clinical presentations and outcome of meningococcal septic shock is closely related to the circulating levels of lipopolysaccharides (LPS) and of *Neisseria meningitidis* DNA (Nm DNA). We have previously explored the distribution of Nm DNA in tissues from large organs of patients dying of meningococcal septic shock and in a porcine meningococcal septic shock model.

**Objective:**

1) To explore the feasibility of measuring LPS levels in tissues from the large organs in patients with meningococcal septic shock and in a porcine meningococcal septic shock model. 2) To evaluate the extent of contamination of non-specific LPS during the preparation of tissue samples.

**Patients and methods:**

Plasma, serum, and fresh frozen (FF) tissue samples from the large organs of three patients with lethal meningococcal septic shock and two patients with lethal pneumococcal disease. Samples from a porcine meningococcal septic shock model were included. Frozen tissue samples were thawed, homogenized, and prepared for quantification of LPS by Pyrochrome^®^ Limulus Amoebocyte Lysate (LAL) assay.

**Results:**

*N. meningitidis* DNA and LPS was detected in FF tissue samples from large organs in all patients with meningococcal septic shock. The lungs are the organs with the highest LPS and Nm DNA concentration followed by the heart in two of the three meningococcal shock patients. Nm DNA was not detected in any plasma or tissue sample from patients with lethal pneumococcal infection. LPS was detected at a low level in all FF tissues from the two patients with lethal pneumococcal disease. The experimental porcine meningococcal septic shock model indicates that also in porcinis the highest LPS and Nm DNA concentration are detected in lungs tissue samples. The quantification analysis showed that the highest concentration of both Nm DNA and LPS are in the organs and not in the circulation of patients with lethal meningococcal septic shock. This was also shown in the experimental porcine meningococcal septic shock model.

**Conclusion:**

Our results suggest that LPS can be quantified in mammalian tissues by using the LAL assay.

## Introduction

*Neisseria meningitidis* (*N. meningitidis*) is a specific human gram-negative diplococcus which primarily resides in the upper airways and occasionally may cause meningitis, septic shock or a combination of meningitis and shock. It may also present as a more lenient invasive infection such as mild or chronic meningococcemia without involvement of the meninges or cause arthritis, pericarditis, panophthalmia or pneumonia (Rosenstein et al., 2001; Stephens et al., 2007). The interaction between the intrusive bacterium and man, causing invasive meningococcal disease (IMD), have been extensively studied and reviewed the last four decades (DeVoe, 1982; van Deuren et al., 2000; Rosenstein et al., 2001; Stephens et al., 2007; Brandtzaeg and van Deuren, 2012; Rouphael and Stephens, 2012). Solitary cases of *N. meningitidis* infections occurs worldwide. Given its propensity to induce clusters of cases, sometimes reaching epidemic proportions, it has long been known as the cause of epidemic meningitis (Weichselbaum, 1887).

The incidence varies over time from region to region and is presently more prevalent in third world countries, particularly in the “meningitis belt” in sub-Saharan Africa (Pardo de Santayana et al., 2023). The ability to cause invasive disease and cluster of cases is related to specific clones of *N. meningitidis.* They are encapsulated (A,B,C,X,Y,W) and reveal specific patterns of house-hold enzymes, outer membrane proteins and lipopolysaccharides (LPS), the latter often denoted lipooligosaccharides (LOS) and may circumvent immune recognition (Caugant and Maiden, 2009; Rouphael and Stephens, 2012; Read, 2014). Meningococcal infection is still a much-feared disease despite the availability of protective vaccines covering the main serogroups A, B, C, Y and W since comparatively few inhabitants in a population are vaccinated. The prevalence is highest among infants and children below 5 years and young adults. However, the disease occurs in all age groups with a higher case fatality rate in patients above 25 years of age (Wang et al., 2019).

In the 1980s it was discovered that the clinical presentations and outcome of IMD were closely associated with the levels of LPS, the endotoxin of *N. meningitidis*, as measured by the Limulus amebocyte lysate (LAL) assay (Harthug et al., 1983; Bjorvatn et al., 1984; Brandtzaeg et al., 1989; Brandtzaeg et al., 1992b; van Deuren et al., 1995; Brandtzaeg et al., 2001a). In the 1990s researchers determined the number of meningococcci per mL plasma or cerebrospinal fluids (CSF) by using real time PCR and relating the numbers per mL to the Glasgow Meningococcal Septicaemia Prognostic Score or the clinical presentation (meningitis, septic shock, a combination of the two, or mild meningococcemia) (Hackett et al., 2002; Ovstebo et al., 2004; Darton et al., 2009). The levels of *N. meningitidis* (Nm-copy number) and the levels of LPS in plasma and CSF were closely related (Ovstebo et al., 2004). More recently the Nm-copy number in tissues from the large organs of patients dying of meningococcal septic shock has been determined and linked to the transcriptomic changes and cytokine levels in tissues from these organs (Hellerud et al., 2015; Brusletto et al., 2017; Brusletto et al., 2020). Subsequently a similar pattern of transcriptomic changes was detected in organ tissues of a porcine meningococcal septic shock model (Hellerud et al., 2015; Brusletto et al., 2022).

LPS and various outer membrane proteins including porins, comprise a large part of the outer leaflet of the outer membrane of the meningococci (Devoe and Gilchrist, 1973; Frasch and Mocca, 1978; Tsai et al., 1981; DeVoe, 1982; Aho, 1989). Furthermore, *N. meningitidis* also produce and secret various proteins including secretory IgA1 protease that possibly may contribute to the invasiveness and host pathology (Tommassen and Arenas, 2017). LPS is, by far, the most potent group of molecules activating various parts of the patients’ immune system and induce clinical symptoms and outcome in a dose dependent manner (Brandtzaeg, 1995; Brandtzaeg et al., 2001a; Brandtzaeg, 2006; Stephens et al., 2007; Ovstebø et al., 2008; Brandtzaeg and van Deuren, 2012). This conclusion is based on multiple measurements of LPS in plasma and cerebrospinal fluid (CSF) by several research groups using the Limulus amebocyte lysate assay (LAL assay) (Harthug et al., 1983; Bjorvatn et al., 1984; Brandtzaeg et al., 1989; Brandtzaeg et al., 1992b; van Deuren et al., 1995; van Deuren et al., 2000; Brandtzaeg et al., 2001a). The high LPS levels in meningococcal patients leading to shock and multiple organ failure have been confirmed to originate from *N. meningitidis* by gas chromatography and mass spectrometry (GC-MS) (Brandtzaeg et al., 1992a). *In vitro* studies of human monocytes and detailed analyses of organ tissues from meningococcal shock patients’ reveal that LPS influence thousands of genes in the lungs and heart, primarily related to inflammation (Ovstebø et al., 2008; Brusletto et al., 2020; Brusletto et al., 2022).

The synthesis, chemistry and functions of *N. meningitidis* LPS have been extensively studied (Kulshin et al., 1992; Pavliak et al., 1993; Kahler and Stephens, 1998; Steeghs et al., 1998; Bjerre et al., 2000; van der Ley et al., 2001; Bjerre et al., 2003; Zughaier et al., 2004). LPS molecules, located in the outer membrane, are translocated by a group of plasma proteins including LPS-binding protein (LPB) and albumin (Gioannini et al., 2002; Post et al., 2005). They are guided to CD14 molecules on immune cells including human monocytes, tissue macrophages, and dendritic cells. Subsequently, single LPS molecules as associated with MD2 and TLR4, are generating transmembrane signals (Bjerre et al., 2003; Gioannini et al., 2004; Yu et al., 2012). The transmembrane signals activates the MyD88-dependent and MyD-independent pathways (Zughaier et al., 2004; Ovstebø et al., 2008).

Although LPS is the most potent group of molecules and carry its name endotoxin rightly, other molecules produced by *N. meningitidis* such as various outer membrane lipoproteins, excreted proteins and fragment of the thin peptidoglycan layer underneath the outer membrane may contribute to the inflammation (Brandtzaeg, 2006; Tommassen and Arenas, 2017). Lipoproteins may activate the human immune system via CD14-TLR2 (Ingalls et al., 2000). DNA from meningococci appear to activate TLR9 (Mogensen et al., 2006). Peptidoglycan fragments may trigger the intracellular located NOD1 and NOD2 receptors (Pridmore et al., 2001; Sprong et al., 2001; Antignac et al., 2003). In summary *in vitro* experiments suggest that these non-LPS molecules may also contribute to gene activation and possibly inflammatory reactions in cases with very high levels of meningococci i.e.10^7^- 10^8^/mL (Hellerud et al., 2008; Ovstebø et al., 2008).

To clarify the contribution of LPS versus non-LPS molecules in details we have developed a porcine model (Hellerud et al., 2010; Hellerud et al., 2015) and a human monocyte target model (Ovstebø et al., 2008). By evaluating the pathophysiological influence of LPS versus non-LPS molecules we have used a wild-type serogroup B reference *N. meningitidis* strain (H44/76) isolated from a patient with lethal septic shock and a mutant of this strain *N. meningitidis* (H44/76lpxA-) completely lacking LPS (Holten, 1979; Steeghs et al., 1998). The results suggest that LPS, by far, is the most potent group of molecules that trigger the immune system and induce the inflammatory response leading to shock and organ dysfunction in a large animal (Hellerud et al., 2010; Hellerud et al., 2015; Hellerud et al., 2017). In subsequent studies we have quantified the levels of *N. meningitidis* in tissues from different larger organs in patients and in the porcine experimental model and measuring the transcriptomic changes and protein levels of key inflammatory molecules (Hellerud et al., 2015; Brusletto et al., 2017; Brusletto et al., 2020; Brusletto et al., 2022).

Since LPS is the most potent group of meningococcal molecules that link whole and disintegrated bacteria and their outer membrane vesicles (blebs) to tissue immune cells, we have asked a key question: Can we measure the levels of LPS in the different tissues using the LAL assay on homogenized tissues dissolved in a LAL assay buffer (Devoe and Gilchrist, 1973; Brandtzaeg et al., 1989; Bjerre et al., 2000; Namork and Brandtzaeg, 2002). Recently published studies suggested that the numbers of meningococci were as high in lung and heart tissue as measured in blood which was reflected in gene activation in both patients and the porcinis (Hellerud et al., 2015; Brusletto et al., 2017; Brusletto et al., 2020; Brusletto et al., 2022). In the present study we have used tissues from three meningococcal septic shock patients as described by Brusletto et al., in 2017 and 2020, to study the usefulness of the LAL assay to detect accumulated LPS in the tissues of different large organs (Hellerud et al., 2015; Brusletto et al., 2017; Brusletto et al., 2020; Brusletto et al., 2022). In addition, we have examined the LPS levels in tissues from the porcine *N. meningitidis* shock experiments (Hellerud et al., 2015). The specimens were treated with sterilized equipment in an effort to avoid major LPS contamination and stored at -80^°^C immediately after the dissection.

We are aware of the possible influence of tissue glycans on the LAL results (Nalepka and Greenfield, 2004). The activation of the LAL assay caused by tissue glycans in orthopedic patients were very low compared with the LPS levels found in the circulation in the three lethal meningococcal patients examined (Nalepka and Greenfield, 2004; Brusletto et al., 2020). We have also examined tissues from patients found dead in bed and examined at Department of Forensic Medicine, University of Oslo. They had positive blood cultures of the gram-positive *Streptococcus pneumoniae* (*S. pneumoniae*) (Hellerud et al., 2015). Previous studies by our group have shown that patients with lethal pneumococcal septic shock and multiple organ failure have a negative LAL assay of heparin plasma (Brandtzaeg et al., 1989).

## Materials and methods

### Bacteria

The international reference strain *N. meningitidis* H44/76, characterized as B;15:P1:7,16:L3,7,9 was used in the porcine experiments (Holten, 1979; Bjune et al., 1991; Piet et al., 2011). In the porcine experiments heath inactivated H44/76 was used for safety reasons (Nielsen et al., 2009).

### Animals

Healthy Norwegian landrace pigs of both sexes with a bodyweight of 30 kg were used in the experiment (Nielsen et al., 2009; Hellerud et al., 2015; Hellerud et al., 2017).

### Experimental design of the porcine study

Eight pigs received exponentially increasing numbers of *N. meningitidis* intravenously by doubling the infusion rate of the bacteria every 30 minutes during 4 h. A total of 5.7x10^10^ meningococci was given to the pig receiving the lowest numbers of bacteria. The successive pigs used in the experiments received two-, three-, four-, five-, six-, 10-, and 20-fold this number of meningococci. Three pigs served as negative controls and received 0.9% NaCl only (Hellerud et al., 2015). After the end of the experiments the animals were euthanized, and biopsies were obtained immediately after from the organs and rapidly frozen on liquid nitrogen. The intention of the experimental model was to simulate the growth of meningococci in the circulation and in large organs as observed in patients (Brandtzaeg and van Deuren, 2012).

### Patients

Three patients (1 up to 18-year-old) with acute lethal meningococcal sepsis and multiple organ failure were included. Leftover, whole blood, serum, and plasma from routine blood sampling was frozen and analyzed later. Organ samples; mainly, lungs, hearts, livers, kidneys, and spleens were collected in parallel with the routine post mortem examination (within 24 h after the patient died), and frozen at −80°C for later analysis (Hellerud et al., 2015; Brusletto et al., 2017; Brusletto et al., 2020; Brusletto et al., 2022).

### Patient controls

Whole blood, and organ samples from two patients dying of microbiologically verified pneumococcal disease (gram-positive infectious control group) were also included in this study to evaluate non-specific LPS contamination during preparation of the tissue samples (Hellerud et al., 2015; Brusletto et al., 2017).

### Autopsy procedure of the patients

The tissue samples have been prepared at the Department of Pathology, Oslo University Hospital, Department of Pathology Stavanger University Hospital, and at the Section for Forensic Pediatric Pathology, Oslo University Hospital, Oslo, Norway (former: Department of Research and Development in Forensic Pathology, The Norwegian Institute of Public Health, Oslo, Norway). All the autopsies have been carried out by pathologists on duty and according to routine procedures, which include sterile equipment for microbiological sampling (Hellerud et al., 2015; Brusletto et al., 2017; Brusletto et al., 2020).

### Homogenization procedure for quantification of LPS in tissue samples

500 µl LAL reagent water (LRW) (Associates of Cape Cod, USA) (LRW contains less than 0.001 EU/mL endotoxin and less than 1.56 pg/mL glucan) was added to a thawed tissue sample previously stored at -80°C, which was trimmed with sterile equipment to weigh approximately 50 mg and then homogenized with Xiril Dispomix (AH diagnostics, Aarhus, Denmark). Each sample was weighed separately and the concentration of LPS calculated per g tissue. After completion, the samples were transferred to Nunc tubes and kept at -80°C until analysis.

### Homogenization procedure for DNA extraction and quantification of *N. meningitidis* DNA in tissue samples

The homogenization procedure and DNA extraction were performed as previously described (Hellerud et al., 2015; Brusletto et al., 2017).

### Bacterial DNA quantification

Bacterial concentrations were determined by bacterial genome DNA quantification utilizing real-time PCR (LightCycler; Roche Diagnostic, Mannheim, Germany) (Ovstebo et al., 2004).

### Quantification of *N. meningitidis* DNA in fresh frozen tissue from patients with lethal systemic meningococcal disease with shock and multiple organ failure and from FF tissue samples from a porcine experimental model

The bacterial load of *N. meningitidis* DNA was examined using quantitative real-time PCR (q-PCR) and primers for the capsule transport A (ctrA) gene (1 copy per *N. meningitidis* DNA) (Frosch et al., 1992; Hackett et al., 2002; Ovstebo et al., 2004; Darton et al., 2009; Hellerud et al., 2015; Brusletto et al., 2017), with a lower detection limit 0.01 pg of genomic DNA (Brusletto et al., 2017). In the human patient study, the results were described as DNA copies/μg human DNA and in the porcine experimental model as DNA copies/g tissue.

### Quantification of *N. meningitidis* LPS and DNA in in circulation from patients with lethal systemic meningococcal disease with shock and multiple organ failure in samples collected on hospital admission

The heparin-blood was collected, centrifuged, plasma pipetted off and aliquoted as described in detail earlier (Brandtzaeg et al., 1989; Brandtzaeg et al., 2001b). Quantification of *N. meningitidis* DNA was performed as earlier described in detail (Ovstebo et al., 2004; Gopinathan et al., 2012). The detection limit was 10^3^ *N. meningitidis* DNA copies/mL. Quantification of LPS in plasma/serum was at first performed with an in house developed Limulus Amebocyte Lysate (LAL) assay and later with Chromo-LAL (Associates of Cape Cod, USA) with a detection limit of 0.2 EU/mL. The serum level is on average 60% of the plasma level for purified *N. meningitidis* LPS (100 EU/mL) (Brandtzaeg et al., 1989; Brandtzaeg et al., 2001b).

### Quantification of LPS in FF tissue samples

Quantification of LPS in the homogenized FF tissue samples were quantified in a Pyrochrome^®^ Limulus Amoebocyte Lysate assay by an endpoint chromogenic method using a diazo-coupling assay kit (Associates of CAPE COD, Inc., Falmouth, MA, USA). Homogenates were diluted in depyrogenated Pyrotube-D^®^ tubes with 500 µl LAL Reagent water (both Associates of CAPE COD, Inc., Liverpool, UK). The diluted samples were heat treated at 75°C for 10 minutes, mixed with pyrochrome solved in Glucashield^®^, a β-D-glucan inhibiting buffer, and incubated in a 96 well Pyroplate^®^ (both Associates of CAPE COD, Inc., Falmouth, MA, USA) on a dry block incubator. Normal human serum was used as negative control and hence as a comparable “background” for the other measurements. After incubation, the procedure was followed according to the instructions from the manufacturer. Detection limit was 3,13 EU/mL.

### Statistical analysis

For correlation analysis, the Spearman procedure in GraphPad Prism Software Version 9.4.1 (GraphPad Software, San Diego, CA, USA) was used.

## Results

### Quantification of *N. meningitidis* DNA and LPS in circulation, and in fresh frozen tissue samples from patients with lethal systemic meningococcal disease with septic shock

The number of *N. meningitidis*/mL in the circulation of the patients with meningococcal septic shock reached from 3.0x 10^7^/mL to 1.0x10^8^/mL (**Table 1**). The LPS levels in heparin plasma or serum ranged from 2140 EU/mL to 3800 EU/mL (**Table 1**) (Brusletto et al., 2017).

**Table 1:** Quantification of *N. meningitidis* (Nm) DNA by q-PCR and LPS by LAL assay method in plasma/serum and in fresh frozen tissue from patients with lethal systemic meningococcal disease with shock and in control patients with microbiologically verified acute lethal pneumococcal disease.

Patient No	Neisserial DNA; copy number of *N. meningitidis/*mL LPS (LAL); EU/mL at admission to hospital * not available	Type of organ tissue	Copies *N. meningitidis* DNA/ug human DNA * not available	LPS (LAL); EU/g tissue * not available
**No 1** **Patient with systemic meningococcal disease (SMD) with shock**	1.0x10^8^copies/mL (serum) 2140 EU/mL (serum) Spinal puncture was performed post mortem. CSF contained 8 EU/mL	Lungs Heart Kidneys Liver Spleen Adrenal gland Duodenum Cerebellum	2.4x10e^8^ 4.2x10e^6^ 6.3x10e^7^ * 5.9x10e^6^ * * *	13400 16200 8500 * 3500 2200 80 800
**No 2** **Patient with systemic meningococcal disease (SMD) with shock**	3.0x10^7^copies/mL (serum) 3800 EU/mL (serum) No spinal puncture was performed	Lungs Heart Kidneys Liver Spleen Adrenal gland Small intestine	2.3x10e^8^ 6.1x10e^7^ 8.3x10e^7^ 1.2x10e^8^ 4.3x10e^7^ * *	23500 5000 1100 4500 5100 2500 3200
**No 3** **Patient with systemic meningococcal disease (SMD) with shock**	* * *	Lungs Heart Kidneys Liver Spleen Adrenal gland	4.1x10e^7^ 3.5x10e^7^ 9.9x10e^6^ 1.7x10e^7^ * *	112100 800 1800 4100 2200 2400
**No 4** **Control patient with lethal pneumococcal disease**	*	Lungs Heart Kidneys Liver Spleen		70 100 70 200 300
**No 5** **Control patient with lethal pneumococcal disease**	*	Lungs Heart Kidneys Liver Spleen Adrenal gland M. vastus med M. vastus lat		590 210 200 500 170 430 240 440

In plasma/serum Nm DNA was measured as copy number of N. meningitidis/mL and LPS as EU/mL. In tissue NmDNA was measured as copies of *N. meningitidis* DNA/ug human DNA and LPS as EU/g tissue. M. vastus med. denotes Musculus vastus medialis and M. vastus lat. denotes Musculus vastus lateralis.

*N. meningitidis* DNA was detected in all FF tissues from patients with lethal shock and multiple organ failure (Brusletto et al., 2017) (**Table 1**). The amount o*f N. meningitidis* DNA in the organs ranged from 5.9 x 10^6^ to 2.4 x 10^8^ copies of *N. meningitidis* DNA/ug human DNA. In general, the highest concentration of *N. meningitidis* DNA was presented in lungs tissue samples from all three patients (**Table 1**).

LPS concentration, quantified as EU/g tissue, was detected in all the patients FF tissue samples (**Table 1**). The amount of LPS in the organs ranged from 80 EU/g tissue (duodenum) to 112,000 EU/g (lung) tissue. In general, the FF tissue samples from lungs revealed the highest concentration and especially patient 3 had an extreme amount of LPS in the lungs (**Table 1**).

In patients with systemic meningococcal disease with shock, the quantification analysis shows in general, that the highest concentration of both *N. meningitidis* DNA and LPS are in the organs and not in the circulation (**Table 1**). The correlation plot for matched pair of data for *N. meningitidis* DNA and LPS concentration for each organ (n=12) showed a weak correlation Spearman r =0,32 (data not shown).

### Quantification of *N. meningitidis* DNA and LPS in circulation, and in fresh frozen tissue samples from control patients with lethal *S. pneumoniae* infection

*N. meningitidis* DNA was not detected in any plasma or tissue sample (Hellerud et al., 2015; Brusletto et al., 2017).

LPS was detected in all FF tissues from the two pneumococcal sepsis patients, shown in **Table 1**. The median amount of LPS in the organs of the controls was 210 EU/g tissue ranging from 70 EU/g tissue to 590 EU/g tissue (**Table 1**). The LPS concentrations were 10-fold to 70-fold higher in the meningococcal patients’ organs than detected in the organs from the control patients infected with *S. pneumoniae*, lacking LPS (**Table 1**).

### Quantification of *N. meningitidis* DNA and LPS in circulation and in fresh frozen tissue samples from porcinis in the porcine experimental model

The median number of *N. meningitidis*/mL in the circulation of the porcinis infused with meningococci was 2x10e^5^ copies/mL (plasma) and the LPS levels in serum was 239 EU/ml (**Table 2**). In the organs, we detected that the median copy number of *N. meningitidis* was 1x10^6^/g tissue in the lungs, 8x10^5^/g in the liver, and below detection limit (10^3^ *N. meningitidis* DNA copies/mL) in the rest of the organs (**Table 2**) (Hellerud et al., 2015).

**Table 2:** Quantification of *N. meningitidis* (Nm) DNA by q-PCR and LPS by LAL method in plasma /serum and fresh frozen tissue from porcinis Nm septic shock experimental model.

Model	Neisserial DNA; median copy number of *N. meningitidis/*mL Median LPS (LAL); EU/mL	Type of organ tissue	Median numbers of *N. meningitidis* DNA/g tissue	Median LPS; EU/g tissue * not available
**Porcine Nm septic shock experimental model** Number of porcinis n =8	2x10e^5^ copies/mL (plasma) 239 EU/ml (serum)	Lungs (n=8) Kidneys (n=8) Liver (n=8) Spleen (n=8)	1.0x10e^6^ <1x10^4^ 8.0x10e^5^ <1x10^4^	26000 * 10000 *

Nm DNA presented as median copy number of *N. meningitidis*/mL and LPS as EU/mL in plasma/serum. In tissue Nm DNA presented as median numbers of *N. meningitidis* DNA/g tissue (detection limit is 1x104) and LPS as EU/g tissue. Detection limit is 1x10^4^.

The LPS concentrations, as quantified as EU/g tissue, were detected in the porcinis FF tissue lung and liver samples (**Table 2**). The LPS concentration in the lungs tissue showed the highest concentration, 26,000 EU/g tissue, while in the liver the LPS concentration was 10,000 EU/g tissue.

The model also showed that the concentration of LPS and *N. meningitidis* DNA are remarkable higher in organs than in the circulation (**Table 2**).

## Discussion

The results from this study suggest that the LPS levels in tissues as the lungs, heart, kidneys, liver, and spleen in the three meningococcal shock patients are higher than we have detected in the circulation of the same patients earlier (Brusletto et al., 2020). Previously, our results suggested that key inflammatory mediators, cytokines, chemokines, and others, were also higher in different organ tissues than in plasma or serum (Hellerud et al., 2015; Brusletto et al., 2020). These results have been reproduced in our porcine shock model (Hellerud et al., 2015). We have hypothesized that key cytokines including TNF, IL-1β, IL-6 and other inflammatory molecules, primarily are generated in the tissues of each organ, induced by accumulating levels of *N. meningitidis* and blebs carrying LPS. The cytokines detected in plasma may be a sort of “overflow” from tissues, but not primarily produced by the leukocytes in the circulation (Brandtzaeg et al., 1996; van Deuren et al., 1998; Gille-Johnson et al., 2012; Hellerud et al., 2015). Also circulating IL-10 is suggested to depress certain leukocyte functions including cytokine production in meningococcal septic shock plasmas (Brandtzaeg et al., 1996; Bjerre et al., 2003). These observations support the hypothesis that circulating cytokines and chemokines primarily are produced in the organ tissues with lower levels of IL-10 than measured in plasma (Hellerud et al., 2015).

Are the high levels of LPS detected in the organs really caused by live or dead meningococci or blebs? Or are the levels of LPS artefacts caused by unspecific LPS contamination during the preparation of the tissues? Our research group has extensive experience with measuring LPS in in different biological specimens collected from patients with lethal meningococcal and pneumococcal septic shock (Brandtzaeg et al., 1989; Brandtzaeg et al., 2001b). To try to answer the question of LPS contamination of the patients’ tissues samples, we have included tissues from patients with acute, lethal *S. pneumoniae* infection (**Table 1**). *S. pneumoniae* is a gram-positive bacterium. It lacks an outer membrane and LPS and does not activate the LAL assay when cultivated on LPS-free media. Plasmas from patients – often splenectomized and unvaccinated - with fulminant pneumococcal sepsis leading to septic shock, multiple organ failure, coagulopathy and death, which is clinically quite similar to fatal meningococcal shock, do not activate the LAL assay (Brandtzaeg et al., 1989). In the forensic tissues samples from patients found dead in bed with microbiologically verified *S. pneumoniae* infection used as controls, we found a comparatively low LAL activity of median 210 EU/g tissue as compared with median 3400 EU/mL in the meningococcal patients’ tissues. Only one tissue (duodenum) from one meningococcal shock patient had a LPS level as low as 80 EU/g. For the rest of the meningococcal patients’ tissue samples there was no overlap of the LPS levels in the tissues as compared with the control patients with lethal pneumococcal infection. The difference in median LPS level between the meningococcal patients’ pulmonary tissue was particularly evident being 70-fold higher than the median control pulmonary tissue of the *S. pneumoniae* control patients.

Although the pathologists and our research group tried to avoid LPS contamination by using sterile equipment and by trimming off tissues during preparation for homogenization, we assume that LPS contamination may have occurred. The small pieces of tissues that was available for LPS measurement made it difficult to cut off all surface areas with sterile scalpels to reduce unspecific LPS contamination. The LPS levels found in the control tissues from the patients with lethal pneumococcal infection were median ten- to seventy-fold lower than those measured in the meningococcal septic shock organs. We also assume that LPS contamination was at the same level in tissues from the three meningococcal sepsis patients as found in the *S. pneumoniae* tissue samples since they were prepared simultaneously by the same people in the same laboratory.

In our LAL assay for detection of endotoxin, a Glucashield^®^ buffer that contains a glucan blocking agent have been used. False positives due to contamination of β-D-glucans will then be ruled out. Also, the use of a diazo-coupling reagent in the LAL assay, that measures optical density (OD) at a higher wavelength will help to avoid false positives due to interference by yellow colored sample from body fluids. We know that human plasma contains inhibitors of the LAL cascade system. These inhibitors are neutralized by heat-inactivation and dilution (Brandtzaeg et al., 1989). The homogenized tissue samples were treated likewise. Other non-LPS tissue components that can influence the LAL assay, has not been identified (Ovstebø et al., 2008).

The correlation plot for matched pair of data for *N. meningitidis* DNA and LPS concentration for each organ (n=12) showed a weak correlation using Spearman r =0,32. One can speculate why this correlation is not stronger. One explanation can be that one meningococcus synthesizes a lot more outer membrane vesicles containing LPS activity than another. The patients were infected with different clones of meningococci. It has been documented that highly virulent meningococci produce multiple outer membrane vesicles (blebs) in patients’ blood (Namork and Brandtzaeg, 2002). Another explanation is methodologically. The high levels of LPS found in the lungs of patients No.3 indicate that the test sample had to be diluted to a greater extent than the other samples. Given our knowledge that *N. meningitidis* LPS *in vivo* is present in different seizes varying from whole bacteria to blebs, the inhomogenous solution could vary in LPS content. We conclude that the high levels of LPS measured in the organs of the fatal meningococcal sepsis patients are valid. The results found in the porcine organs support our conclusion (**Table 2**). The lungs of the meningococcal patients had the highest levels of LPS per gram tissue. The same was observed in the porcinis. The bacterium accumulated rapidly in the pigs’ lung and liver but was below detection limit in the other organs. The high levels of *N. meningitidis* DNA and LPS found in these tissues presumably were a consequence of active transport from the circulation to the tissues of the organs. We assume that upregulated phagocytosis by lung macrophages plays an important role although the infusion site in the pigs may play a role in the pulmonary accumulation (Nielsen et al., 2009).

A second question that arises from our results is: Are different tissues in the large organs also an important proliferation site of *N. meningitidis* in patients developing septic shock and multiple organ failure and not primarily the circulating blood? The question has arisen since our previous studies suggested that only 1 in 1000 circulating *N. meningitidis* were cultivable in blood from the shock patients (Ovstebo et al., 2004). The numbers of circulating meningococci were determined by realtime -PCR and compared with growth of colony forming units on direct plating of serial diluted blood samples collected before initiation of antibiotic treatment (Brandtzaeg et al., 1989; Ovstebo et al., 2004; Brandtzaeg, 2006). Previous attempts to quantify the levels of circulating meningococci by direct plating have clearly underestimated the magnitude of the bacteremia with exception of one shock patient described by Zwahlen et al. (La Scolea et al., 1981; Zwahlen and Waldvogel, 1984; Sullivan and LaScolea, 1987). We know that meningococci are present and alive in the extravascular space in patients’ skin as observed with direct microscopy of biopsies and by needle aspiration and cultivation 13 hours after initiation of intravenous antibiotic treatment (van Deuren et al., 1993; Harrison et al., 2002). Given the observations from the porcine experiments that non-viable meningococci may accumulate in the lungs and liver, the high levels of LPS in the human tissues may be caused by active transfer of viable and possible non-viable meningococci from circulation to the extravascular space in the lungs and other organs in patients. We conclude that the high tissue levels of LPS, particularly in the lungs of patients, is caused by active transfer of live and dead bacteria and blebs from circulating blood but may also be the result of active proliferation varying from tissue to tissue. We assume that upregulated phagocytosis by local and transient macrophages may play a role of this phenomenon if it is conferred by future research. The question of local tissue proliferation of *N. meningitidis* in large organs remains a hypothesis.

## Conclusion

In conclusion, our results suggest that LPS can be measured in mammalian tissues by using the LAL assay. The lungs are the organs with the highest LPS levels followed by the heart in two of the three meningococcal shock patients. Contamination of tissues with non-specific LPS is a major challenge when using LAL as the assay method. To estimate the amount of LPS contamination during the preparation of our material we have used tissues from patients with lethal pneumococcal infection. These controls suggest that less than ten percent of the LAL activity can be ascribed to contamination. In pigs, infused with increasing doses of heath inactivated meningococci, the LAL activity in the tissues increased markedly and were much higher than found in circulation suggesting a rapid accumulation in the extravascular space in the lungs and liver. In future quantitative studies of LPS in organs from meningococcal and other patients, more focus must be given to meticulously handling of the specimens and by using LPS-free instruments at every step. Removing surface tissue layers will be necessary since these layers are presumably always contaminated during the dissection and preparation procedures. LAL reagents, not reacting with tissue glucans, should be used. Furthermore, in future research the true nature of meningococcal LPS can be documented by determining the levels of 3-OH-12:0 (three hydroxy lauric acid) as a marker of neisserial lipid A (Brandtzaeg et al., 1992a). This fatty acid is uncommon in the lipid A part of the LPS molecule from most gram-negative bacteria and will support the conclusions obtained by the LAL assay. Unfortunately, we did not have enough tissue material to verify our result by this method.

## Data availability statement

The original contributions presented in the study are included in the article/supplementary material. Further inquiries can be directed to the corresponding author.

## Ethics statement

The studies involving human participants, were approved by the Regional Medical Ethical Committee of South East Norway (2011/1413C “Translational research, meningococcal disease” and 2011/753 “Studies of invasive meningococcal and pneumococcal disease”), (Biobank 948). The patients’ samples were collected after informed consent from patient parents or relatives and according to the Helsinki declaration. The Director of Public Prosecutions approved the use of forensic material for this research. The studies were conducted in accordance with the local legislation and institutional requirements. Written informed consent for participation in this study was provided by the participants’ legal guardians/next of kin. The animal study was approved by The animal study was approved by the Norwegian Animal Research Authority, and animals were treated according to the Norwegian Laboratory Animal Regulations. The porcine experiments were performed in accordance with the Norwegian laboratory animal regulations and the University Animal Care Committee approved the protocol. The study was conducted in accordance with the local legislation and institutional requirements. Written informed consent was obtained from the minor(s)’ legal guardian/next of kin for the publication of any potentially identifiable images or data included in this article.

## Author contributions

BB: Conceptualization, Data curation, Formal Analysis, Investigation, Methodology, Project administration, Software, Visualization, Writing – original draft, Writing – review & editing, Resources, Validation. BH: Conceptualization, Investigation, Methodology, Writing – review & editing, Data curation, Resources, Funding acquisition. RØ: Formal Analysis, Funding acquisition, Investigation, Methodology, Resources, Software, Writing – review & editing, Data curation. PB: Conceptualization, Data curation, Funding acquisition, Investigation, Methodology, Resources, Supervision, Validation, Writing – original draft, Writing – review & editing.

## References

[B1] Aho E. L. (1989). Molecular biology of class 5 outer membrane proteins of *Neisseria meningitidis* . Microb. Pathog. 7 (4), 249–253. doi: 10.1016/0882-4010(89)90043-0 2516217

[B2] Antignac A. Rousselle J. C. Namane A. Labigne A. Taha M. K. Boneca I. G. (2003). Detailed structural analysis of the peptidoglycan of the human pathogen *Neisseria meningitidis* . J. Biol. Chem. 278 (34), 31521–31528. doi: 10.1074/jbc.M304749200 12799361

[B3] Bjerre A. Brusletto B. Ovstebo R. Joo G. B. Kierulf P. Brandtzaeg P. (2003). Identification of meningococcal LPS as a major monocyte activator in IL-10 depleted shock plasmas and CSF by blocking the CD14-TLR4 receptor complex. J. Endotoxin Res. 9 (3), 155–163. doi: 10.1179/096805103125001559 12831456

[B4] Bjerre A. Brusletto B. Rosenqvist E. Namork E. Kierulf P. Øvstebø R.. (2000). Cellular activating properties and morphology of membrane-bound and purified meningococcal lipopolysaccharide. J. Endotoxin Res. 6 (6), 437–445. doi: 10.1177/09680519000060060501 11521068

[B5] Bjorvatn B. Bjertnaes L. Fadnes H. O. Flaegstad T. Gutteberg T. J. Kristiansen B. E.. (1984). Meningococcal septicaemia treated with combined plasmapheresis and leucapheresis or with blood exchange. Br. Med. J. (Clin Res. Ed) 288 (6415), 439–441. doi: 10.1136/bmj.288.6415.439 PMC1444687 6419956

[B6] Bjune G. Høiby E. A. Grønnesby J. K. Arnesen O. Fredriksen J. H. Halstensen A.. (1991). Effect of outer membrane vesicle vaccine against group B meningococcal disease in Norway. Lancet 338 (8775), 1093–1096. doi: 10.1016/0140-6736(91)91961-s 1682541

[B7] Brandtzaeg P. (1995). “Pathogenesis of meningococcal infections,” in Meningococcal disease. Ed. Cartwright. K. (Chichester, UK: John Wiley & Sons), 71–114.

[B8] Brandtzaeg P. (2006). “Pathogenesis and pathophysiology of invasive meningococcal disease,” in Handbook of Meningococcal Disease: Infection Biology, Vaccination, Clinical Management. Eds. Frosch M. Maiden. M. C. J. (Weinheim, Germany: Wiley-VCH Verlag GmbH & Co), 427–480.

[B9] Brandtzaeg P. Bjerre A. Ovstebo R. Brusletto B. Joo G. B. Kierulf P. (2001a). *Neisseria meningitidis* lipopolysaccharides in human pathology. J. Endotoxin Res. 7 (6), 401–420. doi: 10.1179/096805101101533016 11753210

[B10] Brandtzaeg P. Bryn K. Kierulf P. Ovstebo R. Namork E. Aase B.. (1992a). Meningococcal endotoxin in lethal septic shock plasma studied by gas chromatography, mass-spectrometry, ultracentrifugation, and electron microscopy. J. Clin. Invest. 89 (3), 816–823. doi: 10.1172/jci115660 1541674 PMC442926

[B11] Brandtzaeg P. Kierulf P. Gaustad P. Skulberg A. Bruun J. N. Halvorsen S.. (1989). Plasma endotoxin as a predictor of multiple organ failure and death in systemic meningococcal disease. J. Infect. Dis. 159 (2), 195–204. doi: 10.1093/infdis/159.2.195 2492587

[B12] Brandtzaeg P. Osnes L. Ovstebo R. Joo G. B. Westvik A. B. Kierulf P. (1996). Net inflammatory capacity of human septic shock plasma evaluated by a monocyte-based target cell assay: identification of interleukin-10 as a major functional deactivator of human monocytes. J. Exp. Med. 184 (1), 51–60. doi: 10.1084/jem.184.1.51 8691149 PMC2192662

[B13] Brandtzaeg P. Ovstebo R. Kierulf P. (2001b). Quantitative detection of bacterial lipopolysaccharides in clinical specimens. Methods Mol. Med. 67, 427–439. doi: 10.1385/1-59259-149-3:427 21337159

[B14] Brandtzaeg P. Ovsteboo R. Kierulf P. (1992b). Compartmentalization of lipopolysaccharide production correlates with clinical presentation in meningococcal disease. J. Infect. Dis. 166 (3), 650–652. doi: 10.1093/infdis/166.3.650 1500752

[B15] Brandtzaeg P. van Deuren M. (2012). Classification and pathogenesis of meningococcal infections. Methods Mol. Biol. 799, 21–35. doi: 10.1007/978-1-61779-346-2_2 21993637

[B16] Brusletto B. S. Hellerud B. C. Loberg E. M. Goverud I. L. Vege A. Berg J. P.. (2017). Traceability and distribution of *Neisseria meningitidis* DNA in archived post mortem tissue samples from patients with systemic meningococcal disease. BMC Clin. Pathol. 17, 10. doi: 10.1186/s12907-017-0049-9 28824331 PMC5559868

[B17] Brusletto B. S. Hellerud B. C. Olstad O. K. Øvstebø R. Brandtzaeg P. (2022). Transcriptomic changes in the large organs in lethal meningococcal shock are reflected in a porcine shock model. Front. Cell Infect. Microbiol. 12. doi: 10.3389/fcimb.2022.908204 PMC9413276 36034711

[B18] Brusletto B. S. Loberg E. M. Hellerud B. C. Goverud I. L. Berg J. P. Olstad O. K.. (2020). Extensive changes in transcriptomic "Fingerprints" and immunological cells in the large organs of patients dying of acute septic shock and multiple organ failure caused by *Neisseria meningitidis* . Front. Cell Infect. Microbiol. 10. doi: 10.3389/fcimb.2020.00042 PMC7045056 32154187

[B19] Caugant D. A. Maiden M. C. (2009). Meningococcal carriage and disease–population biology and evolution. Vaccine 27 Suppl 2 (4), B64–B70. doi: 10.1016/j.vaccine.2009.04.061 19464092 PMC2719693

[B20] Darton T. Guiver M. Naylor S. Jack D. L. Kaczmarski E. B. Borrow R.. (2009). Severity of meningococcal disease associated with genomic bacterial load. Clin. Infect. Dis. 48 (5), 587–594. doi: 10.1086/596707 19191644

[B21] DeVoe I. W. (1982). The meningococcus and mechanisms of pathogenicity. Microbiol. Rev. 46 (2), 162–190. doi: 10.1128/mr.46.2.162-190.1982 6126800 PMC281537

[B22] Devoe I. W. Gilchrist J. E. (1973). Release of endotoxin in the form of cell wall blebs during in *vitro* growth of *Neisseria meningitidis* . J. Exp. Med. 138 (5), 1156–1167. doi: 10.1084/jem.138.5.1156 4200775 PMC2139435

[B23] Frasch C. E. Mocca L. F. (1978). Heat-modifiable outer membrane proteins of *Neisseria meningitidis* and their organization within the membrane. J. Bacteriol 136 (3), 1127–1134. doi: 10.1128/jb.136.3.1127-1134.1978 102633 PMC218548

[B24] Frosch M. Muller D. Bousset K. Muller A. (1992). Conserved outer membrane protein of *Neisseria meningitidis* involved in capsule expression. Infect. Immun. 60 (3), 798–803. doi: 10.1128/iai.60.3.798-803.1992 1371768 PMC257557

[B25] Gille-Johnson P. Smedman C. Gudmundsdotter L. Somell A. Nihlmark K. Paulie S.. (2012). Circulating monocytes are not the major source of plasma cytokines in patients with sepsis. Shock 38 (6), 577–583. doi: 10.1097/SHK.0b013e3182746e52 23143060

[B26] Gioannini T. L. Teghanemt A. Zhang D. Coussens N. P. Dockstader W. Ramaswamy S.. (2004). Isolation of an endotoxin-MD-2 complex that produces Toll-like receptor 4-dependent cell activation at picomolar concentrations. Proc. Natl. Acad. Sci. U.S.A. 101 (12), 4186–4191. doi: 10.1073/pnas.0306906101 15010525 PMC384716

[B27] Gioannini T. L. Zhang D. Teghanemt A. Weiss J. P. (2002). An essential role for albumin in the interaction of endotoxin with lipopolysaccharide-binding protein and sCD14 and resultant cell activation*. J. Biol. Chem. 277 (49), 47818–47825. doi: 10.1074/jbc.M206404200 12372833

[B28] Gopinathan U. Ovstebo R. Olstad O. K. Brusletto B. Dalsbotten Aass H. C. Kierulf P.. (2012). Global effect of interleukin-10 on the transcriptional profile induced by *Neisseria meningitidis* in human monocytes. Infect. Immun. 80 (11), 4046–4054. doi: 10.1128/iai.00386-12 22966040 PMC3486056

[B29] Hackett S. J. Guiver M. Marsh J. Sills J. A. Thomson A. P. Kaczmarski E. B.. (2002). Meningococcal bacterial DNA load at presentation correlates with disease severity. Arch. Dis. Child 86 (1), 44–46. doi: 10.1136/adc.86.1.44 11806883 PMC1719043

[B30] Harrison O. B. Robertson B. D. Faust S. N. Jepson M. A. Goldin R. D. Levin M.. (2002). Analysis of pathogen-host cell interactions in purpura fulminans: expression of capsule, type IV pili, and PorA by *Neisseria meningitidis* in *vivo* . Infect. Immun. 70 (9), 5193–5201. doi: 10.1128/iai.70.9.5193-5201.2002 12183570 PMC128269

[B31] Harthug S. Bjorvatn B. Osterud B. (1983). Quantitation of endotoxin in blood from patients with meningococcal disease using a limulus lysate test in combination with chromogenic substrate. Infection 11 (4), 192–195. doi: 10.1007/bf01641194 6352508

[B32] Hellerud B. C. Nielsen E. W. Thorgersen E. B. Lindstad J. K. Pharo A. Tonnessen T. I.. (2010). Dissecting the effects of lipopolysaccharides from nonlipopolysaccharide molecules in experimental porcine meningococcal sepsis. Crit. Care Med. 38 (6), 1467–1474. doi: 10.1097/CCM.0b013e3181de8c94 20400898

[B33] Hellerud B. C. Olstad O. K. Nielsen E. W. Troseid A. M. Skadberg O. Thorgersen E. B.. (2015). Massive organ inflammation in experimental and in clinical meningococcal septic shock. Shock 44 (5), 458–469. doi: 10.1097/shk.0000000000000441 26473439

[B34] Hellerud B. C. Orrem H. L. Dybwik K. Pischke S. E. Baratt-Due A. Castellheim A.. (2017). Combined inhibition of C5 and CD14 efficiently attenuated the inflammatory response in a porcine model of meningococcal sepsis. J. Intensive Care 5, 21. doi: 10.1186/s40560-017-0217-0 28261486 PMC5327570

[B35] Hellerud B. C. Stenvik J. Espevik T. Lambris J. D. Mollnes T. E. Brandtzaeg P. (2008). Stages of meningococcal sepsis simulated in *vitro*, with emphasis on complement and Toll-like receptor activation. Infect. Immun. 76 (9), 4183–4189. doi: 10.1128/iai.00195-08 18591229 PMC2519403

[B36] Holten E. (1979). Serotypes of *Neisseria meningitidis* isolated from patients in Norway during the first six months of 1978. J. Clin. Microbiol. 9 (2), 186–188. doi: 10.1128/jcm.9.2.186-188.1979 107188 PMC272987

[B37] Ingalls R. R. Lien E. Golenbock D. T. (2000). Differential roles of TLR2 and TLR4 in the host response to Gram-negative bacteria: lessons from a lipopolysaccharide-deficient mutant of *Neisseria meningitidis* . J. Endotoxin Res. 6 (5), 411–415. doi: 10.1177/09680519000060050301 11521065

[B38] Kahler C. M. Stephens D. S. (1998). Genetic basis for biosynthesis, structure, and function of meningococcal lipooligosaccharide (endotoxin). Crit. Rev. Microbiol. 24 (4), 281–334. doi: 10.1080/10408419891294216 9887366

[B39] Kulshin V. A. Zähringer U. Lindner B. Frasch C. E. Tsai C. M. Dmitriev B. A.. (1992). Structural characterization of the lipid A component of pathogenic *Neisseria meningitidis* . J. Bacteriol 174 (6), 1793–1800. doi: 10.1128/jb.174.6.1793-1800.1992 1548229 PMC205780

[B40] La Scolea L. J. Jr. Dryja D. Sullivan T. D. Mosovich L. Ellerstein N. Neter E. (1981). Diagnosis of bacteremia in children by quantitative direct plating and a radiometric procedure. J. Clin. Microbiol. 13 (3), 478–482. doi: 10.1128/jcm.13.3.478-482.1981 6787069 PMC273818

[B41] Mogensen T. H. Paludan S. R. Kilian M. Ostergaard L. (2006). Live Streptococcus pneumoniae, Haemophilus influenzae, and *Neisseria meningitidis* activate the inflammatory response through Toll-like receptors 2, 4, and 9 in species-specific patterns. J. Leukoc. Biol. 80 (2), 267–277. doi: 10.1189/jlb.1105626 16731773

[B42] Nalepka J. L. Greenfield E. M. (2004). Detection of bacterial endotoxin in human tissues. Biotechniques 37 (3), 413–417. doi: 10.2144/04373st06 15470896

[B43] Namork E. Brandtzaeg P. (2002). Fatal meningococcal septicaemia with "blebbing" meningococcus. Lancet 360 (9347), 1741. doi: 10.1016/s0140-6736(02)11721-1 12480427

[B44] Nielsen E. W. Hellerud B. C. Thorgersen E. B. Castellheim A. Pharo A. Lindstad J.. (2009). A new dynamic porcine model of meningococcal shock. Shock 32 (3), 302–309. doi: 10.1097/SHK.0b013e31819c37be 19174740

[B45] Ovstebo R. Brandtzaeg P. Brusletto B. Haug K. B. Lande K. Hoiby E. A.. (2004). Use of robotized DNA isolation and real-time PCR to quantify and identify close correlation between levels of *Neisseria meningitidis* DNA and lipopolysaccharides in plasma and cerebrospinal fluid from patients with systemic meningococcal disease. J. Clin. Microbiol. 42 (7), 2980–2987. doi: 10.1128/jcm.42.7.2980-2987.2004 15243048 PMC446236

[B46] Ovstebø R. Olstad O. K. Brusletto B. Møller A. S. Aase A. Haug K. B.. (2008). Identification of genes particularly sensitive to lipopolysaccharide (LPS) in human monocytes induced by wild-type versus LPS-deficient *Neisseria meningitidis* strains. Infect. Immun. 76 (6), 2685–2695. doi: 10.1128/iai.01625-07 18362127 PMC2423066

[B47] Pardo de Santayana C. Tin Tin Htar M. Findlow J. Balmer P. (2023). Epidemiology of invasive meningococcal disease worldwide from 2010-2019: a literature review. Epidemiol. Infect. 151, e57. doi: 10.1017/s0950268823000328 37052295 PMC10126893

[B48] Pavliak V. Brisson J. R. Michon F. Uhrín D. Jennings H. J. (1993). Structure of the sialylated L3 lipopolysaccharide of *Neisseria meningitidis* . J. Biol. Chem. 268 (19), 14146–14152. doi: 10.1016/S0021-9258(19)85220-1 8314780

[B49] Piet J. R. Huis in 't Veld R. A. van Schaik B. D. van Kampen A. H. Baas F. van de Beek D.. (2011). Genome sequence of *Neisseria meningitidis* serogroup B strain H44/76. J. Bacteriol 193 (9), 2371–2372. doi: 10.1128/jb.01331-10 21378179 PMC3133077

[B50] Post D. M. B. Zhang D. Eastvold J. S. Teghanemt A. Gibson B. W. Weiss J. P. (2005). Biochemical and functional characterization of membrane blebs purified from *Neisseria meningitidis* serogroup B*. J. Biol. Chem. 280 (46), 38383–38394. doi: 10.1074/jbc.M508063200 16103114

[B51] Pridmore A. C. Wyllie D. H. Abdillahi F. Steeghs L. van der Ley P. Dower S. K.. (2001). A lipopolysaccharide-deficient mutant of *Neisseria meningitidis* elicits attenuated cytokine release by human macrophages and signals via toll-like receptor (TLR) 2 but not *via* TLR4/MD2. J. Infect. Dis. 183 (1), 89–96. doi: 10.1086/317647 11076707

[B52] Read R. C. (2014). *Neisseria meningitidis*; clones, carriage, and disease. Clin. Microbiol. Infect. 20 (5), 391–395. doi: 10.1111/1469-0691.12647 24766477

[B53] Rosenstein N. E. Perkins B. A. Stephens D. S. Popovic T. Hughes J. M. (2001). Meningococcal disease. N Engl. J. Med. 344 (18), 1378–1388. doi: 10.1056/nejm200105033441807 11333996

[B54] Rouphael N. G. Stephens D. S. (2012). *Neisseria meningitidis*: biology, microbiology, and epidemiology. Methods Mol. Biol. 799, 1–20. doi: 10.1007/978-1-61779-346-2_1 21993636 PMC4349422

[B55] Sprong T. Stikkelbroeck N. van der Ley P. Steeghs L. van Alphen L. Klein N.. (2001). Contributions of *Neisseria meningitidis* LPS and non-LPS to proinflammatory cytokine response. J. Leukoc. Biol. 70 (2), 283–288. doi: 10.1189/jlb.70.2.283 11493621

[B56] Steeghs L. den Hartog R. den Boer A. Zomer B. Roholl P. van der Ley P. (1998). Meningitis bacterium is viable without endotoxin. Nature 392 (6675), 449–450. doi: 10.1038/33046 9548250

[B57] Stephens D. S. Greenwood B. Brandtzaeg P. (2007). Epidemic meningitis, meningococcaemia, and *Neisseria meningitidis* . Lancet 369 (9580), 2196–2210. doi: 10.1016/s0140-6736(07)61016-2 17604802

[B58] Sullivan T. D. LaScolea L. J. Jr. (1987). *Neisseria meningitidis* bacteremia in children: quantitation of bacteremia and spontaneous clinical recovery without antibiotic therapy. Pediatrics 80 (1), 63–67. doi: 10.1542/peds.80.1.63 3110730

[B59] Tommassen J. Arenas J. (2017). Biological functions of the secretome of *Neisseria meningitidis* . Front. Cell Infect. Microbiol. 7. doi: 10.3389/fcimb.2017.00256 PMC5472700 28670572

[B60] Tsai C. M. Frasch C. E. Mocca L. F. (1981). Five structural classes of major outer membrane proteins in *Neisseria meningitidis* . J. Bacteriol 146 (1), 69–78. doi: 10.1128/jb.146.1.69-78.1981 6783622 PMC217053

[B61] van der Ley P. Steeghs L. Hamstra H. J. ten Hove J. Zomer B. van Alphen L. (2001). Modification of lipid A biosynthesis in *Neisseria meningitidis* lpxL mutants: influence on lipopolysaccharide structure, toxicity, and adjuvant activity. Infect. Immun. 69 (10), 5981–5990. doi: 10.1128/iai.69.10.5981-5990.2001 11553534 PMC98725

[B62] van Deuren M. Brandtzaeg P. van der Meer J. W. (2000). Update on meningococcal disease with emphasis on pathogenesis and clinical management. Clin. Microbiol. Rev. 13 (1), 144–166. doi: 10.1128/CMR.13.1.144 10627495 PMC88937

[B63] van Deuren M. Netea M. G. Hijmans A. Demacker P. N. Neeleman C. Sauerwein R. W.. (1998). Posttranscriptional down-regulation of tumor necrosis factor-alpha and interleukin-1beta production in acute meningococcal infections. J. Infect. Dis. 177 (5), 1401–1405. doi: 10.1086/517824 9593034

[B64] van Deuren M. van der Ven-Jongekrijg J. Bartelink A. K. van Dalen R. Sauerwein R. W. van der Meer J. W. (1995). Correlation between proinflammatory cytokines and antiinflammatory mediators and the severity of disease in meningococcal infections. J. Infect. Dis. 172 (2), 433–439. doi: 10.1093/infdis/172.2.433 7622886

[B65] van Deuren M. van Dijke B. J. Koopman R. J. Horrevorts A. M. Meis J. F. Santman F. W.. (1993). Rapid diagnosis of acute meningococcal infections by needle aspiration or biopsy of skin lesions. Bmj 306 (6887), 1229–1232. doi: 10.1136/bmj.306.6887.1229 7684633 PMC1677561

[B66] Wang B. Santoreneos R. Giles L. Haji Ali Afzali H. Marshall H. (2019). Case fatality rates of invasive meningococcal disease by serogroup and age: A systematic review and meta-analysis. Vaccine 37 (21), 2768–2782. doi: 10.1016/j.vaccine.2019.04.020 30987851

[B67] Weichselbaum A. (1887). Ueber die Aetiologie der akuten Meningitis cerebro-spinalis. Fortschr. der Med. 5, 573–583.

[B68] Yu L. Phillips R. L. Zhang D. Teghanemt A. Weiss J. P. Gioannini T. L. (2012). NMR studies of hexaacylated endotoxin bound to wild-type and F126A mutant MD-2 and MD-2·TLR4 ectodomain complexes. J. Biol. Chem. 287 (20), 16346–16355. doi: 10.1074/jbc.M112.343467 22433852 PMC3351295

[B69] Zughaier S. M. Tzeng Y. L. Zimmer S. M. Datta A. Carlson R. W. Stephens D. S. (2004). *Neisseria meningitidis* lipooligosaccharide structure-dependent activation of the macrophage CD14/Toll-like receptor 4 pathway. Infect. Immun. 72 (1), 371–380. doi: 10.1128/iai.72.1.371-380.2004 14688118 PMC343956

[B70] Zwahlen A. Waldvogel F. A. (1984). Magnitude of bacteremia and complement activation during *Neisseria meningitidis* infection: study of two co-primary cases with different clinical presentations. Eur. J. Clin. Microbiol. 3 (5), 439–441. doi: 10.1007/bf02017367 6437810

